# Tm^3+^; Yb^3+^:Zn_2_TiO_4_ near infrared to blue upconversion phosphors for anti-counterfeit applications

**DOI:** 10.1039/d3ra03238h

**Published:** 2023-08-04

**Authors:** Joydip Dutta, Mitesh Chakraborty, Vineet Kumar Rai

**Affiliations:** a Central Research Facility, Indian Institute of Technology (Indian School of Mines) Dhanbad 826004 Jharkhand India; b Department of Physics, St. Xavier's College Ranchi 834001 Jharkhand India miteshsxc@gmail.com; c Laser and Spectroscopy Laboratory, Department of Applied Physics, Indian Institute of Technology (Indian School of Mines) Dhanbad 826004 Jharkhand India vineetkrrai@iitism.ac.in

## Abstract

Tm^3+^; Yb^3+^:Zn_2_TiO_4_ samples have been synthesized using a solid state reaction route. The phase, lattice parameters, crystallite size has been examined using X-ray Diffraction (XRD) and high resolution transmission electron microscopy (HRTEM). An intense peak of Yb^3+^ codoped samples is observed near ∼957 nm due to the ^2^F_7/2_ → ^2^F_5/2_ transition in diffuse reflectance spectra (DRS), which confirms the presence of Yb^3+^ ion in the prepared compound. The optical band gap of Yb^3+^ codoped samples has been calculated using Kubelka–Munk function. The Raman spectra corresponds to incorporation of Tm^3+^/Yb^3+^ at the octahedral and tetrahedral site of the spinel host. The emission spectra recorded by using 370 nm excitation wavelength shows intense blue colour band corresponding to the ^1^G_4_ → ^3^H_6_ transition of Tm^3+^ ion. The upconversion (UC) emission spectra recorded by using 980 nm laser excitation source shows emission bands due to the ^1^G_4_ → ^3^H_6_, ^1^G_4_ → ^3^F_4_ and ^3^H_4_ → ^3^H_6_ transitions of Tm^3+^ ion in the host matrix lying in the blue, red and NIR regions respectively. There is effective enhancement of about ∼35 times in the blue UC emission intensity with incorporation of Yb^3+^ at 3% doping concentration in the prepared sample. The anti-counterfeit application of the optimized upconverting phosphor has been successfully demonstrated.

## Abstract 2

The upconversion (UC) emission spectra recorded by using 980 nm laser of the prepared phosphors. The excitation wavelength shows intense blue colour. The anti-counterfeit application of the prepared sample demonstrated.

## Graphical abstract

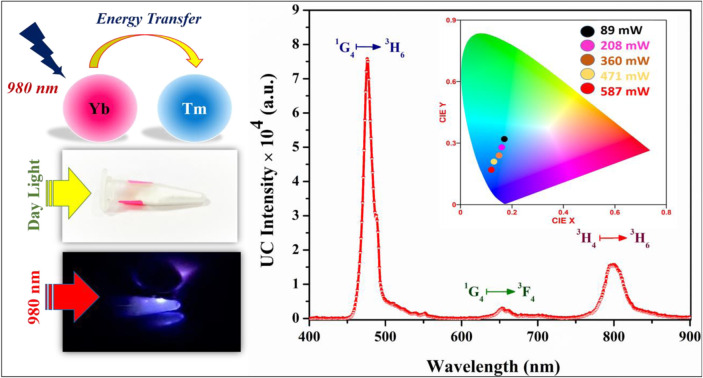

## 1 Introduction

Studies of rare earths (REs) doped phosphors have gained considerable attention among material scientists due to their diversified applications in the field of *viz.* opto electronic devices, light emitting diodes, optical temperature sensors, high efficiency solar cells, anti-counterfeit security applications, finger print detection, *etc.*^1–5^ As compared to research in organic luminescent materials, RE doped non-carbon oxides have several advantages *viz.* large Raman shifts, high optical stability, sharp emission spectra and cost-effective.^6–8^ Doping RE in suitable luminescent hosts is always preferred for their multicolour emission and colour tuning property.^9,10^ Also, the lanthanide ions because of their large electronic configuration, consist of many energy states, some of which have longer lifetime, hence by using the different external excitation sources, a series of optical transitions can be monitored.^11,12^

The luminescent property of a material is investigated by optical mechanism of upconversion (UC) and downshifting (DS). UC is a non-linear optical process to create high energy photons using excitation from two or more lower energy photons. DS is the reverse process of UC in which a high frequency photon is converted into low frequency photons.^3,4^ Through literature survey, it has been noticed that emission intensity of upconverting materials can be improved by codoping with suitable sensitizers.^13,14^ One of the sensitizer widely used by researchers is Yb^3+^ ion. Due to small shielding of the outer electrons, Yb^3+^ ions strongly interact with neighbouring ions and the absorption cross-section of Yb^3+^ ions in the near-infrared source is large, hence it easily transfers energy to activator ions in the host matrix.^15,16^

Nowadays, anti-counterfeit technology is widely used in classified documents, currency notes, identity cards verification, *etc.*^17,18^ These applications are very relevant in public offices and business organizations.^3^ Near infrared to visible luminescent materials explored as anti-counterfeit security ink are more reliable and difficult to replicate. Since upconverting materials can be designed to produce selective emission colours by suitable choice of host and dopants.^19–21^

Among the suitable hosts, Zn_2_TiO_4_ have inverse spinel structure with the standard chemical formula B_2_AO_4_. It can also be written as B_T_(AB)_O_O_4_, where T in the subscript denotes tetrahedral sites and O signifies octahedral sites. Half of Zn^2+^ cations and all of Ti^4+^ cations occupy octahedral sites and another half of Zn^2+^ cations occupy tetrahedral sites. O^2−^ is an oxygen anion having six and four coordination with the cations in the base compound.^22^ The physical and electrical properties of Zn_2_TiO_4_ have been investigated by various researchers.^23,24^ But, studies of REs doped Zn_2_TiO_4_ as luminescent material for applications in NIR to efficient blue frequency upconversion and anti-counterfeit is for the first time to the best of our knowledge.

In the present work, Tm^3+^, Yb^3+^:Zn_2_TiO_4_ luminescent materials have been synthesized by using the solid state reaction technique. The structural properties of the prepared samples have been explored using X-ray diffraction (XRD), High resolution transmission electron microscopy (HRTEM), Raman and diffuse-reflectance spectroscopy. The effect of Yb^3+^ codoping on UC and DS has been studied using UV and near-infrared excitation sources. The vacant states created due to doping and codoping of RE^3+^ ions (Tm^3+^; Yb^3+^) at the A-site and B-site of the spinel have been studied using electron paramagnetic resonance (EPR) spectroscopy. The anti-counterfeit security application has been demonstrated in the present investigation.

## 2 Experimental

### 2.1 Materials and methods for Zn_2_TiO_4_:Tm^3+^, Yb^3+^ synthesis

The Tm^3+^, Yb^3+^:Zn_2_TiO_4_ inverse spinel materials have been synthesized by using solid state reaction method. The precursors ZnO, TiO_2_, Tm_2_O_3_ and Yb_2_O_3_ were mixed in stoichiometric proportion in agate mortar for 2 hours. The host raw materials *i.e.* ZnO and TiO_2_ have been taken from Merck (99.0% purity) and the dopants Tm_2_O_3_ and Yb_2_O_3_ from Sigma Aldrich (99.9% purity). The homogeneous mixture was kept in the alumina crucible and the samples were annealed in an electric furnace at 800 °C for 24 hours. The annealed samples were again grind in the agate mortar for 30 minutes and used for structural, luminescence and EPR studies. The balanced chemical reaction is presented as follows:1
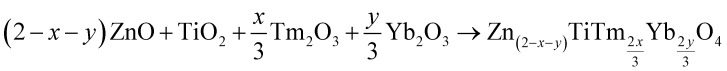


For the synthesis of codoped Zn_2_TiO_4_ crystals, Tm_2_O_3_ (*x*) optimized is fixed at 1 mol% and Yb_2_O_3_ (*y*) is varied as 1 mol%, 3 mol% and 5 mol%.

### 2.2 Measurements and characterization

The X-ray diffraction (XRD) patterns of the samples were recorded using Rigaku D/Max 2200 with Cu K_α_ radiation (*λ* = 1.5406 Å) in the diffraction angle range of 20° to 80° at a scanning rate of 0.02° min^−1^. The surface morphology and particle size of the doped/codope Zn_2_TiO_4_ phosphors compound has been analyzed using high resolution transmission electron microscopy (HR-TEM) JEM-2100F. The energy dispersive X-ray (EDX) measurement has been recorded using Field emission scanning electron microscopy (FESEM) Supra 55 with an air lock chamber. The diffuse reflectance spectra of the prepared phosphors have been analysed employing Cary series (5000) UV-vis-NIR spectrophotometer. UC emission study has been performed with a Princeton triple turret grating monochromator associated with a photomultiplier tube (PMT) upon excitation with a 980 nm continuous wave (CW) laser diode. The emission and excitation spectra are recorded by using a ‘Lifetime Spectrometer’ upon excitation with a Xenon arc lamp. Raman spectroscopy analysis has been carried out by Horiba scientific (LabRAM HR-UV-Open) Raman Spectrometer. EPR spectrum of the prepared phosphors has been performed by a conventional Bruker EMX spectrometer operating at X-band frequency of 9.446 GHz. All characterizations have been performed at room temperature (∼28 °C). The lifetime measurement was recorded by Horiba PTI Quanta-master for the study of decay curves.

## 3 Results and discussion

### 3.1 XRD studies

**Fig. 1:**
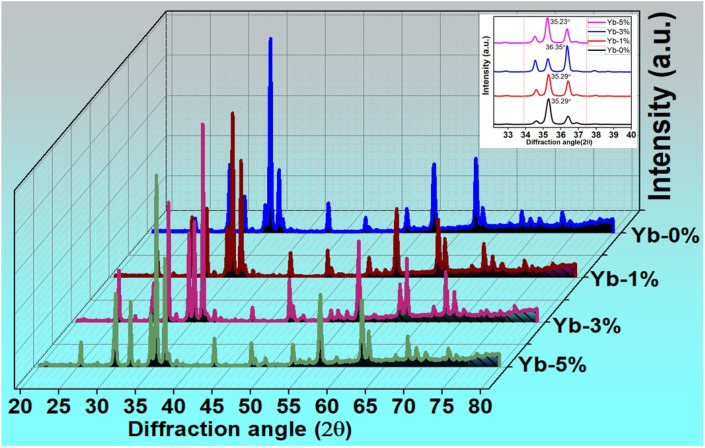
XRD patterns of Tm^3+^/Yb^3+^ codoped Zn_2_TiO_4_ phosphors. The inset shows the diffraction peak shift with increase in doping concentration of Yb^3+^ ion in the host.

The phase and lattice parameters of the prepared phosphors 
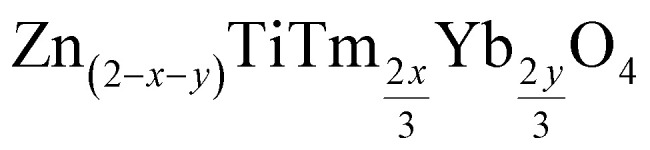
 have been investigated using XRD at room temperature. Fig. 1 depicts the XRD patterns of the prepared phosphors corresponding to Xpert High Score plus reference no. 98-010-9093 of tetragonal phase with space group *P*4_1_22 and space group number 91. The most prominent peak observed for Yb-0%, Yb-1%, Yb-3% and Yb-5% near 35.29°, 35.29°, 36.35° and 35.23° respectively. No individual peaks of Tm_2_O_3_ and Yb_2_O_3_ are observed in the XRD study of the prepared samples. This indicates single tetragonal phase of the prepared compound. The inset of Fig. 1 shows the shift in the most intense Bragg's diffraction angle (2*θ*) with increase in doping of Yb^3+^ ions in the host matrix. The shift in XRD peak and change in lattice parameters with incorporation of Yb^3+^ ion in the Zn_2_TiO_4_:Tm^3+^ is due to difference in ionic radius of Yb^3+^ (0.97 Å) and Zn^2+^ (0.74 Å). Though the ionic radius of Tm^3+^ (0.96 Å) is comparable to Yb^3+^, the increase in doping concentration of Yb^3+^ is attributed to lattice strain for the substitutional occupancy of Yb^3+^ ion in the tetrahedral and octahedral site of Zn^2+^ in the host. The calculated lattice parameters of the prepared samples are presented in Table 1. The crystallite size of the prepared samples are calculated using Scherrer formula^25^2
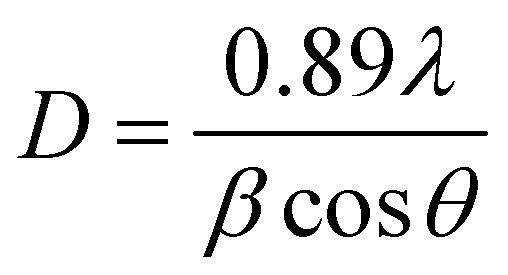
where, *λ* = 0.154 nm is the wavelength of Cu Kα radiation, *θ* denotes the Bragg's diffraction angle with respect to crystal plane and *β* is the full-width at half maximum for specific diffraction peak (Table 1). The crystallite sizes, calculated from Hall–Williamson (WH) relation,^26^ are 32.71 nm, 36.45 nm, 41.67 nm, 32.14 nm for Zn_2_TiO_4_:Tm^3+^ (1%) and Zn_2_TiO_4_:Tm^3+^(1%), Yb^3+^(0%, 1%, 3%, 5%) samples.

**Table 1:** Lattice parameters, energy band gap within the parenthesis and average crystallite size for the optimized doped/codoped Zn_2_TiO_4_:Tm^3+^/Yb^3+^ phosphors

Phosphors	*a* (Å)	*c* (Å)	Cell volume (A^3^)	Av. cryst. size (nm)
Zn_1.99_TiTm_0.0067_O_4_ (3.88 eV)	6.042	8.409	306.977	32.21
Zn_1.98_TiTm_0.0067_Yb_0.0067_O_4_ (3.93 eV)	5.962	8.422	299.364	36.03
Zn_1.96_TiTm_0.0067_Yb_0.02_O_4_ (3.78 eV)	5.993	8.416	302.276	41.24
Zn_1.94_TiTm_0.0067_Yb_0.033_O_4_ (3.86 eV)	6.003	8.377	301.874	32.50

It is clear from Table 1 that the average crystallite size increases with increase in doping concentration of Yb^3+^ from 0% to 3% and again decreases for 5% doping. The change in volume of the unit cell is related to density and optical transparency of the crystal.^27^ The difference in cationic radius of Yb^3+^ and Zn^2+^ ions produces internal strain and this may shift crystal boundaries which rearranges the grain size of the system and alters the crystallite structure of the prepared phosphors.

### 3.2 Surface morphological and EDX study

**Fig. 2:**
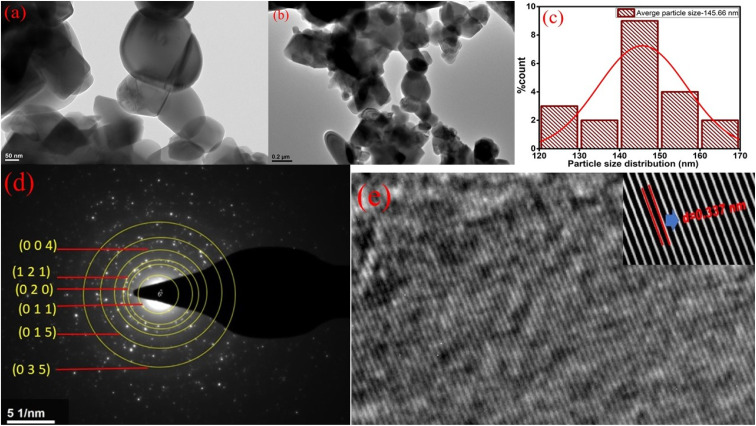
HR-TEM images of the optimized sample Zn_1.96_TiTm_0.0067_Yb_0.02_O_4_ in (a) 50 nm (b) 0.2 μm resolution (c) Histogram depicts the particle size distribution (d) SAED pattern with miller indices labelled across different planes (e) FFT pattern representing *d*-spacing of the (1 2 1) plane.

**Fig. 3:**
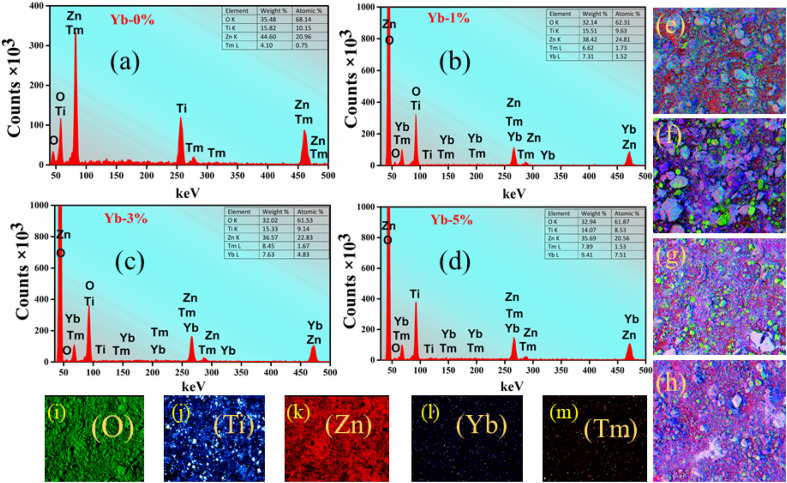
(a)–(d): EDX spectrum of the prepared phosphors; (e)–(h): corresponding EDX colour mapping; (i)–(m): elemental distribution represented in parenthesis of the prepared phosphors Zn_2_TiO_4_:Tm^3+^, Yb^3+^.

The surface morphology of the optimized sample Zn_1.96_TiTm_0.0067_Yb_0.02_O_4_ (Zn_2_TiO_4_:Tm^3+^-1%/Yb^3+^-3%) is presented in Fig. 2. The high resolution transmission electron microscopy (HR-TEM) images in two scales are depicted in Fig. 2 (a) 50 nm (b) 0.2 μm. It is clear from these images that some dark spot is observed which confirms the presence of dopants Tm^3+^ ions and Yb^3+^ ions in the prepared compound. Fig. 2c shows the particle size distribution of the optimized sample. The average particle size calculated using image J software employing normal distribution in histogram is 145.66 nm. The crystallinity of the prepared phosphor is investigated from selected area electron diffraction (SAED) pattern shown in Fig. 2d. The circular white spots corresponding to different diameters of the circle is formed by the electron beam's diffraction at regular lattice points of the unit cell. The miller indices of the different planes of the crystal structure are labelled in Fig. 2d. The miller indices calculated using image J software resembles XRD analysis discussed in the section 3.1. The *d*-spacing of the plane (1 2 1) evaluated using integral transformation property of direct and inverse fast Fourier transform (FFT) in the SAED pattern illustrated in Fig. 2e is 3.37 Å. The elements present in the prepared phosphors can be detected by studying EDX spectrum. The EDX spectra, colour mapping of the corresponding spectra and elemental distribution of the prepared phosphors Zn_2_TiO_4_:Tm^3+^-1% (a) Yb^3+^-0% (b) Yb^3+^-1% (c) Yb^3+^-3% and (d) Yb^3+^-5% is presented in Fig. 3. It is evident that Zn, Ti, Tm, Yb and O elements are present in the doped/codoped phosphors. The inset table in Fig. 3a–d reflects the elemental compositions in wt% and at% observed in the scanning region of the EDX instrument. It is evident from at% values shown in the inset of Fig. 3b–d that Tm : Yb are nearly of the ratios 1 : 1, 1 : 3 and 1 : 5 respectively which confirms the homogeneity and molar mixing composition of the dopants/codopants in the host.

### 3.3 Optical diffuse reflectance and Raman analysis

**Fig. 4:**
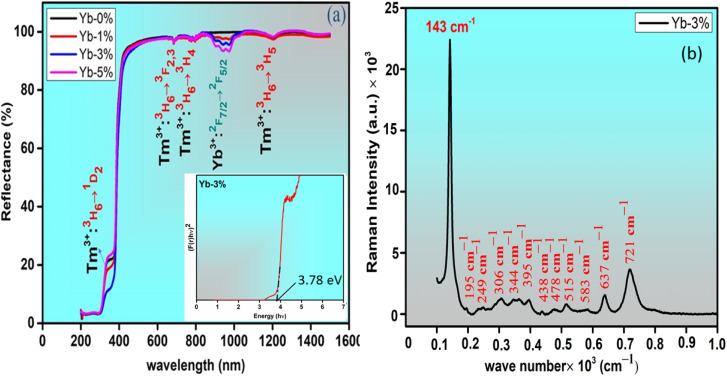
(a) Diffuse reflectance spectra of (i) Zn_2_TiO_4_:Tm^3+^-1%, Yb^3+^-0% (ii) Zn_2_TiO_4_:Tm^3+^-1%, Yb^3+^-1% (iii) Zn_2_TiO_4_:Tm^3+^-1%, Yb^3+^-3% (iv) Zn_2_TiO_4_:Tm^3+^-1%, Yb^3+^-5%. The inset shows the optical band gap of the optimized phosphors Zn_1.96_TiTm_0.0067_Yb_0.02_O_4_ (Yb-3%) using Kubelka–Munk function (b) Raman spectrum of the optimized sample.

The diffuse reflectance spectra (DRS) of the prepared phosphors are recorded in the range of 200–1600 nm with respect to the reference of BaSO_4_ (Fig. 4a). In the prepared phosphors peak bands for Tm^3+^ ion are observed at ∼296 nm, ∼688 nm, ∼787 nm and ∼1202 nm corresponding to the ^3^H_6_ → ^1^D_2_, ^3^H_6_ → ^3^F_2,3_, ^3^H_6_ → ^3^H_4_ and ^3^H_6_ → ^3^H_5_ transitions respectively.^28^ An intense peak of Yb^3+^ ion in the codoped samples is observed in Fig. 3a((ii), (iii) and (iv)) near ∼957 nm due to the ^2^F_7/2_ → ^2^F_5/2_ transition.^29^

The optical band gap of the Yb^3+^ doped and undoped Zn_2_TiO_4_:Tm^3+^ phosphor can be evaluated from the DRS spectra using the well-known Kubelka–Munk (KM) theory.^30^ The KM function *F*(*R*_∞_)*i.e.* the reflectance with respect to a reference for each observed wavelength can be mathematically expressed as,3
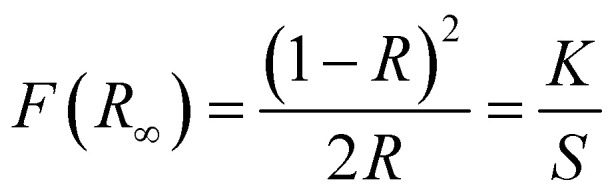
where, ‘*R*’ is the diffuse reflectance ratio of the observed spectrum, ‘*K*’ is the absorption coefficient and ‘*S*’ denotes coefficient of scattering. The well known Tauc relation^31^ used to evaluate the optical band gap ‘*E*_g_’ is given by,

4 *αhν* = *A*(*hν* − *E*_g_)^*n*^

where, ‘*α*’ is the absorption coefficient. ‘*A*' is the constant of proportionality and ‘*hν*’ is the energy of incident light in eV, ‘*n*' is the exponential constant which depends upon the nature of electronic transitions. Using eqn (3) and (4), the diffuse reflectance spectrum can be expressed in terms of following KM function *F*(*R*_∞_),

5 [*hνF*(*R*_∞_)]^2^ = *A*(*hν* − *E*_g_)

Using eqn (5), the band gaps *E*_g_ for (i) Zn_2_TiO_4_:Tm^3+^-1%, Yb^3+^-0% (ii) Zn_2_TiO_4_:Tm^3+^-1%, Yb^3+^-1% (iii) Zn_2_TiO_4_:Tm^3+^-1%, Yb^3+^-3% (iv) Zn_2_TiO_4_:Tm^3+^-1%, Yb^3+^-5% are 3.88 eV, 3.93 eV, 3.78 eV and 3.86 eV respectively (Table 1). It is clear from Table 1 that there is no direct correlation between average crystallite size and energy band gap of the prepared samples. The decrease in band gap is related to increase in crystallinity of the prepared samples and increase in band gap may be due to higher values of direct and/or indirect band gap of Yb_2_O_3_.^31^ This may be due to interstitial occupancy or agglomeration of Yb^3+^ ion about the grain boundary in the host matrix. This is consistent with the observations of the HR-TEM images presented in different resolutions in Fig. 2.

The Raman intensity as a function of Raman frequency (cm^−1^) of the optimized sample Zn_1.96_TiTm_0.0067_Yb_0.02_O_4_ is illustrated in Fig. 4b. The intensity is plotted in the range of 100 to 1200 cm^−1^. The inverse spinel zinc titanate of the form B_2_AO_4_ has the chemical formula Zn_2_TiO_4_ which can also be written as [(ZnTi)ZnO_4_] with 56 atom unit cell. All Zn ions and half of Ti ions within the parenthesis occupy octahedral site and other half of Ti ions occupy tetrahedral site.

The most intense mode near ∼143 cm^−1^ is due to B_1g_ mode of rutile TiO_2_.^32^ A low intense mode near ∼195 cm^−1^ may be due to translation motion/free rotation of Tm^3+^ ion at the octahedral site of the crystal structure.^33^ The E_g_ mode near ∼306 cm^−1^ may be due to vibration of TiO_6_/ZnO_6_ which signifies local lattice effects in the octahedral sites.^34^ The intense peak near ∼438 cm^−1^ is due to E_2_ mode of ZnO.^32^ Similarly, the peak at ∼583 cm^−1^ is due to E_1_ mode of ZnO.^32^ The peak near ∼438 cm^−1^ is attributed to A_1g_ mode of tetragonal Zn_2_TiO_4_. It corresponds to vibration of ZnO_4_ mode which reflects the local lattice effects in the tetrahedral sublattice.^32,34^ The peaks near (395, 515, 637) cm^−1^ is related to Tm–O stretching.^33^ Unlike in other Raman studies, the peak of B_2g_ mode of rutile TiO_2_ near ∼826 cm^−1^ is absent in the present investigation. The different Raman active modes observed in the present study of the optimized sample confirms the incorporation of Tm^3+^/Yb^3+^ in the host Zn_2_TiO_4_.

### 3.4 Photoluminescence and EPR studies

**Fig. 5:**
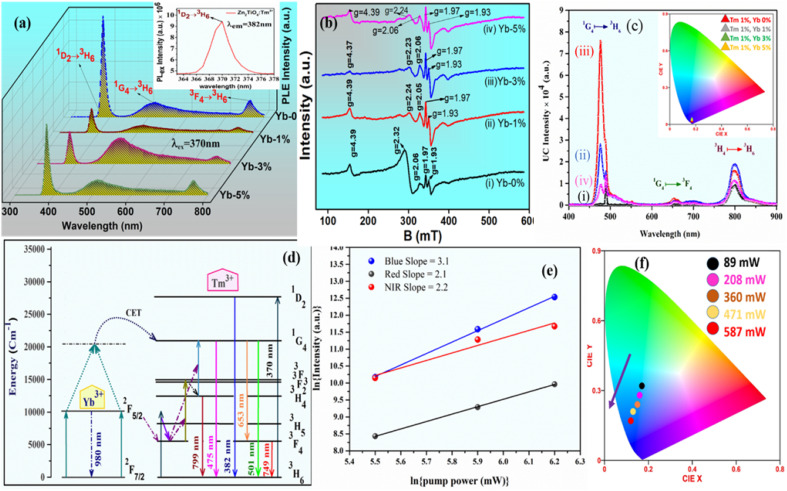
(a) PL emission spectra at 370 nm excitation wavelength. The inset represents the excitation spectrum of Zn_2_TiO_4_:Tm^3+^ phosphor at 382 nm emission. (b) and (c) represents the EPR intensity and UC emission spectra of the powder samples (i) Zn_2_TiO_4_:Tm^3+^-1%, Yb^3+^-0% (ii) Zn_2_TiO_4_:Tm^3+^-1%, Yb^3+^-1% (iii) Zn_2_TiO_4_:Tm^3+^-1%, Yb^3+^-3% (iv) Zn_2_TiO_4_:Tm^3+^-1%, Yb^3+^-5% respectively recorded at room temperature. The inset of (c) shows the CIE plot of UC emission intensity. (d) Schematic energy level diagram (e) pump power dependence study of Tm^3+^-Yb^3+^ codoped Zn_2_TiO_4_ phosphors (f) The corresponding CIE plot of the pump power variation in the optimized sample.

The photoluminescence (PL) emission spectra of the prepared phosphors at (i) Zn_2_TiO_4_:Tm^3+^-1%, Yb^3+^-0% (ii) Zn_2_TiO_4_:Tm^3+^-1%, Yb^3+^-1% (iii) Zn_2_TiO_4_:Tm^3+^-1%, Yb^3+^-3% (iv) Zn_2_TiO_4_:Tm^3+^-1%, Yb^3+^-5% observed at excitation wavelength ∼370 nm is depicted in Fig. 5a. The emission spectra exhibit peaks near ∼382 nm, ∼501 nm and ∼749 nm corresponding to the ^1^D_2_ → ^3^H_6_, ^1^G_4_ → ^3^H_6_ and ^3^F_4_ → ^3^H_6_ transitions of the Tm^3+^ ion.^35^ The excitation spectra consists of band peaking near ∼370 nm related to the ^1^D_2_ → ^3^H_6_ transition.^35^ The maximum photoluminescence emission (PLE) intensity is observed for Tm-1%, Yb-0% sample. This confirms self-quenching of the activator Tm^3+^ ions in the host. There can be various reasons for quenching phenomenon in the present investigation.^36^

(i) The ionic radius of Tm^3+^ is comparable with Yb^3+^ ion. With increase in doping concentration of Yb^3+^ ion from 0% to 5%, electron captured zinc vacancies are created in the host matrix. The well known phenomenon of self-trapping of electrons may be induced to maintain charge neutrality of the compound which ultimately decrease the PLE intensity.3Zn^2+^ → Tm^3+^ + Yb^3+^ + V_Zn_^2+^

(ii) The non-radiative energy transfer among Yb^3+^–Yb^3+^ ions due to exchange interaction, radiative reabsorption or multipole–multipole interaction adversely affect the PLE intensity.

The critical distance (*R*_c_) evaluated using Blasse equation^36^ is found to be 29.99 Å. This value of *R*_c_ indicates multipolar interaction. Further, after using the Dexter and Schulman theory to investigate the type of multipolar interaction,^2,36^ the value of *q* obtained from graphical interpretation is about ∼7.6 which is near to 8. This shows that the dipole–quadrupole interaction is dominant among Yb^3+^ ions.

Fig. 5b shows the EPR spectra of the powder samples Zn_2_TiO_4_:Tm^3+^/Yb^3+^ recorded at room temperature. The EPR spectra of Zn_2_TiO_4_:Tm^3+^ phosphors exhibit a number of resonance signals of varying intensity with effective *g*-values near ∼4.39, 2.32, 2.06, 1.97 and 1.93 shown in Fig. 5b(i). With incorporation of Yb^3+^ ion in the host, shift of resonant peaks is noticed attributed to *g*-values near ∼4.39, 2.24, 2.05. No change has been seen in the position of EPR peaks for *g* ∼ 1.97 and 1.93. Interestingly there is no change in peak position with increase in doping concentration of Yb^3+^ ion from 1 mol% to 5 mol%. It is ascertain from Fig. 4b that there are large number of resonance signals with different *g*-values due to diversified crystal field interaction of the rare earth (RE) dopants Tm^3+^/Yb^3+^ at different site-symmetry with different coordination numbers in the host.^37^ The EPR signals of Tm^3+^/Yb^3+^ ions remain unresolved at room temperature due to short spin–lattice relaxation time.^38^ Hence all resonance signals observed in Fig. 5b are related to zinc and oxygen vacancies created by RE dopants at the Zn-site of the inverse spinel structure Zn_2_TiO_4_. Further, the peak shift observed in Fig. 5b(ii) is only for *g* values greater than 2. This is due to increase in Zn vacancies with doping of Yb^3+^ ion at the Zn site of the host, which modifies the crystal field interaction of the RE dopants and/or exchange interaction of Yb^3+^–Yb^3+^ ions in the host matrix.^39–41^ The *g* values less than 2 are related to oxygen vacancies.^42^ There is no evidence of dipolar broadening with increase in doping concentration of Yb^3+^ ions in the host. This is coexistent with the experimental findings of the PL study discussed earlier. There is strong correlation between *g* values near ∼4.39 with the average crystallite size calculated in Table 1. The resonant intensity of the EPR signals decrease at this *g*-value from Yb-0% to Yb-3% and again starts to increase for Yb-5%, similar to the change in crystallite size with doping concentration of Yb^3+^ ion. As the crystallite size increases, there is decrement in *d*-spacing and EPR signal intensity also follows the same. This is consistent with the structural studies discussed in Section 3.1, that with increase in average crystallite size, relative concentration of vacancies decreases.^43^

### 3.5 Upconversion, pump power studies, lifetime measurements and demonstration of security ink for anti-counterfeit applications

The upconversion (UC) emission spectra for all the developed Zn_2_TiO_4_:Tm^3+^, Yb^3+^ phosphors upon 980 nm laser diode excitation have been recorded and represented in Fig. 4c. For all the prepared doped and codoped phosphors three UC emission bands peaking at ∼476 nm (blue), ∼662 nm(red), ∼799 nm (NIR) corresponding to the ^1^G_4_ → ^3^H_6_, ^1^G_4_ → ^3^F_4_ and ^3^H_4_ → ^3^H_6_ transitions respectively have been observed in the 400–900 nm wavelength range.^35^ It is worthwhile to mention that the intense blue UC emission coming from the Tm^3+^–Yb^3+^ codoped phosphors can be clearly visible in the natural environment. From Fig. 5c, it is clear that the blue UC emission band for Zn_2_TiO_4_:Tm^3+^-1%, Yb^3+^-3% codoped phosphors is more intense as compared to the other doped/codoped phosphors.

An improvement in the blue UC emission intensity around ∼35 times for Zn_2_TiO_4_:Tm^3+^-1%, Yb^3+^-3% as compared to Zn_2_TiO_4_:Tm^3+^-1%, Yb^3+^-0% codoped phosphors has been observed in the present investigation. No additional peaks were observed in the emission study on the codoping of Yb^3+^ ions to the doped Zn_2_TiO_4_:Tm^3+^ ions system. The strongest blue UC emission produced by Zn_2_TiO_4_:Tm^3+^-1%, Yb^3+^-3% phosphors is due to the better environment produced by the Zn_2_TiO_4_ host around the dopants Tm^3+^, Yb^3+^ which avoid quenching process by preventing the creation of large number of RE ions clusters.^5,35,44^ This is compatible with the EPR investigation Fig. 5b reported in Section 3.4 of the present study. The schematic energy level diagram for the Tm^3+^-Yb^3+^ ions to describe the complete information about the UC emission mechanism is shown in Fig. 5d. In the Tm^3+^-Yb^3+^ codoped system upon 980 nm laser excitation; Yb^3+^ ion effectively absorbs 980 nm photons. Since the Yb^3+^ ion have large absorption cross-section corresponding to the ^2^F_7/2_ → ^2^F_5/2_ transition, it can effectively transfer its excitation energy to the Tm^3+^ ion through cooperative energy transfer (CET) process.^44^ The Yb^3+^ ions after absorbing the 980 nm photons are excited from the ground state ^2^F_7/2_ to the excited state ^2^F_5/2_. These excited Yb^3+^ ions transfers their excitation energy to the Tm^3+^ ions through ground state absorption (GSA). The Tm^3+^ ion present in the ^3^F_4_ state through excited state absorption (ESA) process gets promoted to ^3^F_2_ and then again to ^1^G_4_ energy levels. A part of the excited ions at ^3^F_2_ energy levels gets depopulated by non-radiative relaxation to the ^3^H_4_ state and then again radiatively relax by emitting a photon to the ground state (^3^H_6_) at wavelength ∼799 nm. Further, a radiative transition of intense blue emission by depopulating the ^1^G_4_ state corresponding to ∼475 nm is observed. Some part of the excited ions at ^1^G_4_ levels also radiatively relax by emitting photon of wavelength ∼653 nm to the ^3^F_4_ state. The Yb^3+^ ion may also absorb the pump excitation energy from 980 nm source and transit upward to a virtual state and then it may transfer the energy to populate the excited state at ^1^G_4_ level of the activator Tm^3+^ ion.

The CIE colour coordinate studies have been done for all the Tm^3+^-Yb^3+^ doped/codoped Zn_2_TiO_4_ phosphors. GoCIE software has been used to evaluate the colour co-ordinates with the help of recorded UC and PL emission spectra. The calculated colour coordinates for all the phosphors are presented in Table 2. All the coordinates of the prepared phosphors lie in the intense blue colour region. The CIE chromaticity diagram study suggests that the Tm^3+^-Yb^3+^ codoped Zn_2_TiO_4_ upconverting phosphors can be applied in blue colour light emitting devices.

**Table 2:** Colour coordinates of the upconversion (UC) and downshifting (DS) fluorescence computed using CIE software of the prepared optical phosphors

Phosphors	Upconversion (UC)		Downshifting (DS)	
	*X*	*Y*	*X*	*Y*
Zn_2_TiO_4_:Tm^3+^-1%, Yb^3+^-0%	0.15	0.31	0.22	0.59
Zn_2_TiO_4_:Tm^3+^-1%, Yb^3+^-1%	0.12	0.16	0.23	0.66
Zn_2_TiO_4_:Tm^3+^-1%, Yb^3+^-3%	0.13	0.19	0.27	0.68
Zn_2_TiO_4_:Tm^3+^-1%, Yb^3+^-5%	0.13	0.23	0.24	0.71

In Tm^3+^; Yb^3+^ codoped Zn_2_TiO_4_ phosphors, three UC emission bands at ∼476 nm (blue), ∼662 nm(red), ∼799 nm (NIR) corresponding to the ^1^G_4_ → ^3^H_6_, ^1^G_4_ → ^3^F_4_, ^3^H_4_ → ^3^H_6_ transitions respectively have been observed. The number of NIR photons responsible for these UC emissions can be evaluated graphically from the slope of the variation of UC intensity with respect to pump power using the well known mathematical relation^45^

6 *I ∝ p*^*n*^

where, ‘*I*’ is the UC emission intensity, ‘*p*’ is the pump power and ‘*n*’ is the number of NIR photons responsible for UC emission.

The slope values for the blue, red and NIR emissions are 3.1, 2.1 and 2.2 respectively and represented in Fig. 5e. In Tm^3+^; Yb^3+^ codoped phosphors, ^3^H_4_ and ^1^G_4_ levels of Tm^3+^ ions are populated by the active participation of two and three NIR photons respectively.^45,46^ It is evident from the chromaticity diagram of Fig. 5f that with increase in pump power the colour coordinate shifts towards the blue region. Hence the prepared upconverting materials can be employed to develop blue emitting display devices.

**Fig. 6:**
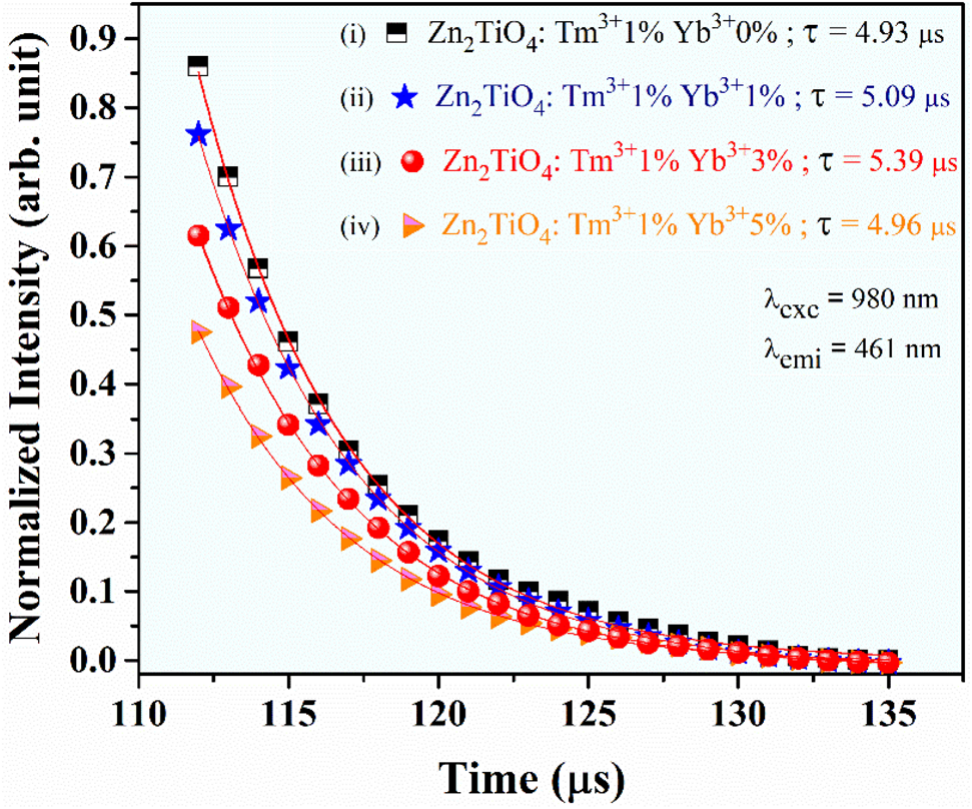
Lifetime spectra of the ^1^G_4_ level of Tm^3+^ for the ^1^G_4_ → ^3^H_6_ (461 nm) transition (*λ*_emi_ = 461 nm) for all the doped/codoped (i) Zn_2_TiO_4_:Tm^3+^-1%, Yb^3+^-0% (ii) Zn_2_TiO_4_:Tm^3+^-1%, Yb^3+^-1% (iii) Zn_2_TiO_4_:Tm^3+^-1%, Yb^3+^-3% (iv) Zn_2_TiO_4_:Tm^3+^-1%, Yb^3+^-5% phosphors at 980 nm pulsed laser radiation.

To monitor the effect of Yb^3+^ ion on the luminescence dynamics, decay time behaviour of the ^1^G_4_ → ^3^H_6_ (461 nm) transition of Tm^3+^ ions for the doped/codoped phosphors has been measured using the 980 nm pulsed laser excitation. Fig. 6 shows the fit of the experimental data to a first order exponential decay equation as^47^7
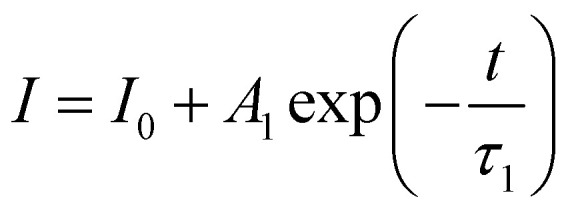
where ‘*I*’ is luminescence intensity at time ‘*t*’ and ‘*I*_0_’ is the initial luminescence intensity, ‘*A*_1_’ is constant and ‘*τ*_1_’ is the decay time.

The decay time of the ^1^G_4_ level corresponding to the ^1^G_4_ → ^3^H_6_ transition of Tm^3+^ ion is ∼4.93 μs, ∼5.09 μs, ∼5.39 μs and ∼4.96 μs for Zn_2_TiO_4_:Tm^3+^-1%; Yb^3+^-0%, Yb^3+^-1%, Yb^3+^ −3% and Yb^3+^-5% respectively. The decay time of the ^1^G_4_ level thus increases with incorporation of Yb^3+^ ion from 1% to 3% doping in the host matrix. This is coexistent with the structural studies (Table 1) and experimental findings of the EPR observations studied in earlier sections. This confirms that along with the energy transfer process the improvement in average crystallite size is also responsible for an enhancement in the emission intensity of the codoped phosphors.^30,47,48^

**Fig. 7:**
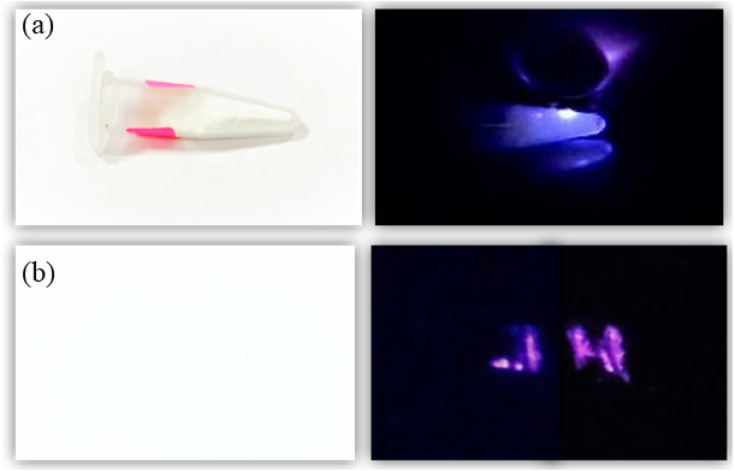
(a) Optical image of the optimized Zn_2_TiO_4_: Tm^3+^-1%, Yb^3+^-3% UC phosphors in day light condition, (b) anti-counterfeit illustration of some classified document coded with ‘JH’ under 980 nm CW laser excitation source.

Further, to demonstrate the anti-counterfeit security ink application, the optimized UC phosphor 20 mg (Tm^3+^, Yb^3+^:Zn_2_TiO_4_) is dissolved in 10 ml ethanol and sonicated at 50 kHz for 2 hours by ultra sonic probe to form good dispersion solution. After that 1 ml of aminopropyltriethoxysilane (APTES) is slowly added to the solution with continuous stirring at 400 rpm, 60 °C for 2 hours and then cooled at RT. After 12 hours aging, the solution was used to write as a security ink (Fig. 7). The letter “JH” which appears to be white is clearly visible upon 980 nm diode laser excitation. Thus, these unique security inks could also be used in printing labels of pharmaceuticals or in printing important documents, other than making our currency notes more secure.

## 4 Conclusion

Tm^3+^; Yb^3+^:Zn_2_TiO_4_ have been successfully synthesized with tetragonal phase by solid state reaction method. The change in average crystallite size of the prepared samples is related to lattice strain developed due to incompatible ionic radii of Tm^3+^/Yb^3+^ and Zn^2+^ ions in the host matrix. The average particle size 145.66 nm has been calculated from the HR-TEM analysis of the prepared phosphors using image J software. Miller index from SAED pattern and *d*-spacing of the optimized sample has been evaluated in the present investigation. An intense peak of Yb^3+^ ions for Yb-codoped samples near ∼957 nm is observed in the DRS spectra which indicate the successful incorporation of Yb^3+^ ion in the host matrix. Self-quenching of the activator Tm^3+^ ions in the host is observed in photoluminescence studies. All resonance signals observed in EPR investigation at room temperature are related to zinc and oxygen vacancies created by Tm^3+^/Yb^3+^ dopants at the Zn-site of the host. Upon excitation with a 980 nm laser CW excitation source, the blue upconversion intensity of Yb^3+^ codoped samples at 3% doping concentration increases several times. The enhancement in intensity has been explained using efficient energy transfer process on codoping of rare-earth ions in the host. The anti-counterfeit application of the prepared sample has been successfully demonstrated. The colour coordinates of the upconverting phosphors indicates blue emission display device applications.

## Author contributions

Joydip Dutta: conceptualization, investigation, data curation, formal analysis, methodology. Mitesh Chakraborty: conceptualization, data curation, formal analysis, investigation, methodology, validation. Vineet Kumar Rai: conceptualization, formal analysis, investigation, supervision, validation, visualization.

## Conflicts of interest

The authors declare no conflict of interest.

## Supplementary Material
